# Investigation of screening questions to identify insomnia in cancer patients

**DOI:** 10.1038/s41598-024-69086-z

**Published:** 2024-08-07

**Authors:** K. Pfeifer, G. Ates, M. Pogorzelski, G. Zaun, A. Rötger, M. Schuler, C. Schöbel, M. Tewes

**Affiliations:** 1 Department of Palliative Medicine, University Hospital Essen, University of Duisburg-Essen, 45147 Essen, Germany; 2 Institute for Digitalization and General Medicine, University Hospital Aachen, Pauwelsstraße 30, 52074 Aachen, Germany; 3 Department of Medical Oncology, West German Cancer Center, University Hospital Essen, University of Duisburg-Essen, 45147 Essen, Germany; 4 Mementor DE, Leipzig, Germany; 5 German Cancer Consortium, Partner Site University Hospital Essen, 45147 Essen, Germany; 6 Center for Sleep- and Telemedicine, West German-Lung-Center at University Hospital Essen, Ruhrlandklinik, 45239 Essen, Germany

**Keywords:** Insomnia, Cancer disease, Screening question, Tiredness, Health care, Oncology, Signs and symptoms

## Abstract

The high prevalence of insomnia in cancer patients leads to a significant reduction in the quality of life of those affected. A detailed record of symptoms therefore plays an essential role for further course of treatment. Which screening instruments enable identification of cancer patients with insomnia is the subject of this single-arm nonrandomized study. During the data collection period, cancer patients meeting the following criteria: self-reported tiredness and/or trouble falling or staying asleep or sleeping too much in an electronic patient-reported outcome measurement were enrolled. For further analysis, focus was placed on the Patient Health Questionnaire Depression Scale (PHQ-8), the Minimal Documentation System (MIDOS^2^) and the Insomnia Severity Index (ISI). Frequency, correlation, and variance analyses were conducted to identify likely predictors of insomnia. Our findings indicate a closer correlation between the screening question pertaining to sleep disorders and the ISI, compared to the question on tiredness and the ISI. The initial recording of sleep-related parameters plays an essential role for cancer patients in order to identify and treat modifiable factors as promptly as possible. For an initial assessment, we recommend asking about trouble falling or staying asleep or sleeping too much.

## Introduction

Insomnia in cancer patients is a common and serious symptom. Studies that focus on the relationship between cancer and insomnia show that 30–50% of respondents report sleeplessness, difficulty sleeping through the night, and daytime sleepiness^1,2^.

Also, a meta-analysis that examined the prevalence of sleep disorders between 1998 and 2021 in cancer patients concluded that, on average, even more than 60% of those experience sleep disorders^3^. While the prevalence of insomnia varies across different entities, it is consistently higher than the average population^1,3^. The concept of insomnia is initially broad and can manifest in a variety of ways: Dominance of difficulty falling asleep or staying asleep, waking up early, and non-restorative sleep^4^.

The European Insomnia Guideline updated in 2023 requires the presence of insomnia to include trouble falling asleep or staying asleep, with resulting daytime sleepiness and impairment of daily living. To be diagnosed, these symptoms need to appear at least several times a week over a period of 3 months^5^.

Patients with insomnia often suffer from somatic and psychological comorbidities, so that a clear trigger often cannot be determined. Especially in patients with advanced cancer disease, multiple factors exist, whether due to the underlying disease per se or in the context of therapy concepts, which lead to an impairment of sleep behavior^4^. The literature consistently shows evidence of a strong association between sleep disorders and cancer disease of various entities^6,7^. There are many reasons for this: the severity of the disease, accompanying anxiety and depression, the side effects of the therapy and medication^8^. In a prospective observational study by Fleming et al., chemotherapy was identified as a main risk factor for persistent insomnia symptoms in patients with breast cancer^9^. Sleep disorders and the resulting daytime tiredness lead to a severely impaired quality of life for patients^2,10^. In a qualitative analysis, Reynolds-Cowie et al. even emphasize that physical and psychological impairments in various areas of life persist long after treatment has completed^11^. In addition, studies point out that sleep disorders receive little attention in doctor-patient conversations because patients fear that the focus may shift from cancer treatment to a less important, incurable comorbidity such as sleep disorders. Moreover, physicians also often have a lack of understanding about addressing sleep disorders as a frequent side effect post cancer diagnosis and treatment^1,4,11,12^. This evidence highlights the importance of a screening procedure implemented in the clinical care routine to identify cancer patients with sleep disorders. Currently, several validated instruments are available for the assessment of insomnia in cancer patients. These include three scales^13–15^, single items^16,17^ and a sleep diary^18^. However, there are references to the fact that these screening practices are not widely used in clinical practice^19^. The Pittsburgh Sleep Quality Index (PSQI) as one of the validated scales provides a comprehensive assessment of sleep quality. Nevertheless, due to its numerous items and complex scoring system, it is rarely used in an oncological setting^20^. In the current practice guideline on insomnia management in cancer patients, the European Society for Medical Oncology (ESMO) additionally emphasizes that there is no standardized instrument that is routinely utilized to assess insomnia in clinical practice^20^. But early detection remains necessary in order to identify modifiable factors as early as possible, which then allows offering patients appropriate treatment options. A systematic literature review by Büttner-Teleagă et al. points to the effectiveness of alternative non-drug treatments of cancer patients with insomnia; especially early interventions with personalized pathways and web-based telehealth programs^21^. Other evidence also suggests that the scope of treatment for insomnia in cancer patients should be expanded to digital therapeutics, parallel with pharmacological options^22^. Advances in digital communication and the increasing presence of digital health applications in everyday clinical practice also open up new treatment concepts for patients, from which they could benefit even more in individual cases, insofar as a symptom burden has been identified by suitable screening tools^21,23^. Patient reported outcome measurement of cancer patients is becoming increasingly important and is firmly anchored in the guidelines for palliative care. At a German comprehensive cancer center (CCC), an electronic palliative medicine-psycho-oncology screening (ePOS) is already being used to identify the symptoms and needs of oncological patients^24,25^. This tool includes validated instruments like Hornheider Screening Instrument (HSI), the Patient Health Questionnaire Depression Scale (PHQ-8), the Generalized Anxiety Disorder (GAD-7) and the Minimal Documentation System (MIDOS^2^)^26–29^. The PHQ-8 includes a question asking the patient about trouble falling or staying asleep or sleeping too much. To avoid overwhelming patients with repetitive questions and thus risking acceptance towards screening methods, our study used this question and the question about tiredness (MIDOS^2^ Questionnaire), both of which are already implemented in ePOS, to check their suitability for detecting insomnia in cancer patients. To our knowledge, the extent to which this screening also enables the identification of cancer patients with sleep disorders has not yet been researched and is therefore the subject of this study.

## Methods

This section outlines the methodological approach of the single-arm non-randomized questionnaire study, which was conducted in 2021 in the outpatient clinic of an oncology center of excellence within a hospital.

### Target and study population

In the outpatient clinic of a German CCC, patients are routinely assessed for symptom burden using ePOS with an electronic device. From January 2021 to December 2021, the insomnia symptom burden data, routinely documented by physicians, was reviewed daily to identify study relevant patients as a first step. This screening procedure was the starting point (t0) for a recruitment of patients. So, all histologically verified cancer patients who reported moderate or severe tiredness (tiredness question) and/or trouble falling or staying asleep or sleeping too much (sleep disorder question) by ePOS (t0) were included in the target population. But subsequently, only those adult patients who visited the outpatient clinic for a second follow-up appointment (t1) and reported again sleep disorder as a symptom burden were asked by the attending physician to participate in the study. Thus, the inclusion criteria were:

- moderate or severe tiredness and/ or trouble falling or staying asleep or sleeping too much on more than half the days or nearly every day at time point t0 and time point t1
- age > 18 years
- sufficient German language competence
- no cognitive impairments

All other patients who did not meet the criteria were excluded.

### Study design

Between January 2021 and December 2021, all patients received verbal information about the study, their data rights, and data safety procedures. After a voluntarily signed consent, participants were asked to independently fill out the validated screening instrument Insomnia Severity Index (ISI) in German. In case of non-participation, treating physicians documented the reasons for non-participation (non-participation survey form). The patients’ supplementary self-completed ISI assessment took place from January 2021 to December 2021. The study was approved by the local ethics committee of the Medical Faculty of University of Duisburg-Essen (20–9793-BO).

### Measurement Instruments

#### ePOS

As mentioned above, ePOS is based on single items of multiple validated screening instruments: HSI, GAD-7, PHQ-8, and MIDOS^2^ to assess the multiple complex needs of cancer patients. The HSI, which demonstrated its validity and internal consistency in a representative study of melanoma patients, is used to examine the psychosocial needs of cancer patients^30^. The GAD-7 is a psychometric instrument designed to identify symptoms of anxiety disorders. According to Löwe et al., who tested the scale in the general population, it has an overall internal consistency of Cronbach’s alpha = 0.89^31^. It is used in the assessment of cancer patients^32^.

To measure the individual parameters, the study’s focus will be on the potential screening items of the PHQ-8 and the MIDOS^2^, as well as the ISI.

#### PHQ-8

The PHQ (Patient Health Questionnaire), comprises 8 questions on depressiveness. Symptoms of major depression are assessed regarding their occurrence within the last two weeks. The third item of the instrument is about trouble falling or staying asleep or sleeping too much. Four values are available for the respondents’ self-report: "Not at all", "Several days", "More than half the days", "Nearly every day”. A scale total of 10 points (maximum value 24 points) indicates depressive symptoms^27^.

Several studies have demonstrated satisfactory internal consistency of the PHQ-8, with Cronbach’s alpha values in the range 0.8 to 0.9^33–35^. A large population-based study by Kroenke et al. demonstrated the criterion and construct validity of the test^27^.

#### MIDOS^2^

The MIDOS^2^, the German version of the Edmonton Symptom Assessment Scale (ESAS), is a validated self- assessment tool to record symptoms. In a list consisting of 10 symptoms (pain, nausea, vomiting, shortness of breath, constipation, weakness, lack of appetite, tiredness, depressiveness, and anxiety), the respondents also mark their individual degree of tiredness using a ranking scale (none, mild, moderate, severe)^29^. MIDOS^2^ has an acceptable Cronbach’s alpha value of between 0.7 and 0.8^36^.

#### Insomnia severity index

The Insomnia Severity Index serves as a validated objective measurement instrument for the assessment of sleep disorders. The validated German form by Dieck et al. includes 7 items to assess the severity, type, and impact of insomnia in the past month. The assessment is based on the fifth edition of the Diagnostic and Statistical Manual of Mental Disorders (DSM- 5) containing diagnostic criteria for difficulty falling asleep and staying asleep. Scoring is based on a five-point Likert scale (0 = none, 4 = very severe), so that a total score of 28 points can be obtained. The exact cut-off values are defined as follows: 0–7: absence of insomnia, 8–14: subthreshold insomnia, 15–21: moderate insomnia, 22–28: severe insomnia. Reliability and validity of ISI has been analyzed in multiple studies^13,37^. Morin et al.^38^ were able to demonstrate satisfactory values regarding internal consistency, as evidenced by Cronbach’s alpha > 0.9. The validated German form, on which the present study is based, also demonstrated adequate internal consistency (Cronbach’s alpha = 0.83)^39^.

#### Data analysis

Data processing and descriptive analyses were performed using SPSS (Statistical Package for the Social Sciences-Version 27/ https://www.ibm.com/de-de/spss). In addition to the screening questions, the pseudonymized data set also includes variables relating to age, sex, and type of cancer (cancer entity, cancer stage). After all variable values are recoded in the same direction; lowest value none and highest value a very strong expression of the symptom load of interest, the summative index “ISI” has been calculated. To calculate the internal consistency of the ISI within the context of this study, Cronbach’s alpha was used^40^. Initial descriptive analyses provide a first insight into the distributions. To analyze sensitivity and specificity of the screening questions we used crosstabs. For the following significance test, a Monte-Carlo simulation with R was applied.

In order to identify suitable predictors of insomnia, frequency and correlation analyses were carried out, as well as an Analysis of Variance (ANOVA) and t-Tests due to the approximately normal distribution of the dependent variable (ISI sum score) to compare the mean values of the inclusion criteria.

For all tests, we defined a significance level of p < 0.05. Boxplots were used for graphical representation. Categories were merged due to small cell occupancies.

The tables contain only valid answers.

### Ethics approval

All procedures performed in studies involving participants were in accordance with the ethical standards of the institutional and local ethical review committee of the Medical Faculty of the University of Duisburg-Essen approved the data analysis (20-9793-BO) and with the 1964 Helsinki declaration and its later amendments or comparable ethical standards.

### Informed consent

Informed consent was obtained from all participants included in the study.

## Results

**Figure 1:**
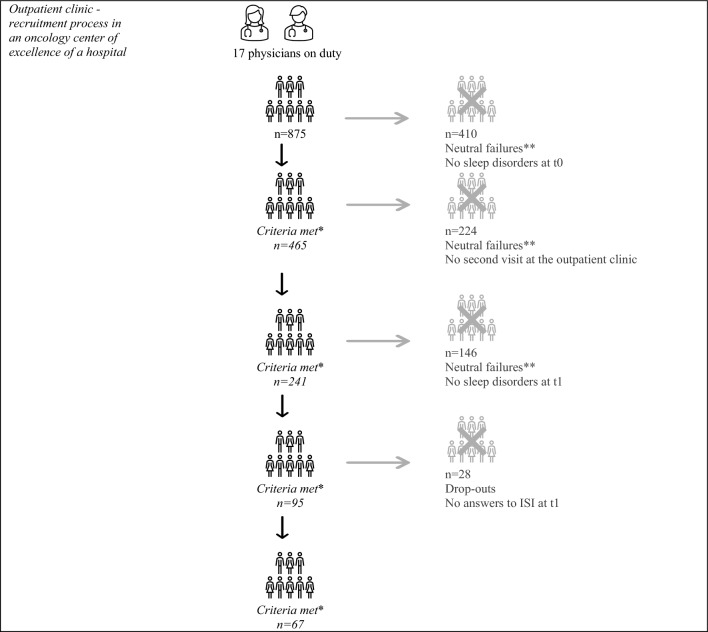
Response rate flowchart. *Inclusion criteria: Histologically verified tumor disease and reported moderate or severe tiredness and/or trouble falling asleep or sleeping too much. **Neutral failure: No subjective sleep disturbance, language barriers, life expectancy < 6 months, excessive symptom burden, cognitive. impairment, due to illness/insufficient symptom control. *Source*: Document analysis of patient data from the period January 2021–December 2021 of a top oncology center; own calculations.

From January 2021 to December 2021, 875 patients indicated whether they experienced tiredness and/or sleep disorder. At t0, 465 patients (53%) met the inclusion criteria. Of these, 92 patients had sleep disorder (value: 2 or 3), 192 patients reported increased tiredness (value: 3 or 4), and 181 patients exhibited both criteria. During the data collection period, 241 patients attended a follow-up appointment (t1) at the outpatient clinic. At t1, all 95 patients still met the criteria and were asked by their oncologist about their interest in participating in this study. Ultimately, 67 patients answered the ISI questions, which corresponds to a response rate of 71% (Fig. 1).

Given the small sample size, the Cronbach’s alpha for the seven-item ISI showed sufficient internal consistency with an acceptable value of 0.73^41^.

### Patient characteristics

The mean age of the participants was 56 years, the gender distribution was almost equal (female 49%, male 51%). Fourteen of the 67 (21%) patients suffered from lung carcinoma and an equal number from gastrointestinal cancer. The majority of patients (78%) were in stage IV according to UICC (Table 1).

**Table 1:** Study participants according to certain characteristics at time t0. *Source*: Document analysis of patient data from the period January 2021–December 2021 of a top oncology center; own calculations.

Characteristic	Target population (n = 67)
Mean age (Standard Deviation)	56 (10) years
Sex n (%)	
Male	34 (51)
Female	33 (49)
Cancer entity n (%)	
Lung cancer	14 (21)
Gastrointestinal cancer	14 (21)
Sarcoma	12 (18)
Urinary tract	4 (6)
Breast cancer	8 (12)
Other	15 (22)
Cancer stage (UICC) n (%)	
0	4 (6)
I	2 (3)
II	3 (4)
III	6 (9)
IV	52 (78)

.

Five of 67 patients did not have sleep disorder at all, 15 on several days, 20 on more than half the days and 27 nearly every day. Four of 66 patients (6%) had no tiredness, 13 patients (20%) had mild tiredness, 33 patients (50%) had moderate tiredness, and 16 patients (24%) had severe tiredness. According to the ISI sum score, 39% had no or subthreshold insomnia, 51% had moderate insomnia, and 10% had severe insomnia (Table 2).

**Table 2:** Frequencies of sleep disorder and tiredness and ISI at t0 and t1.

	t0	t1
Sleep disorder n (%)		Not available
Not at all	5 (8)	
Several days	15 (22)	
More than half the days	20 (30)	
Nearly every day	27 (18)	
n	67	
Tiredness n (%)		Not available
None	4 (6)	
Light	13 (20)	
Medium	33 (50)	
Strong	16 (24)	
n	66	
ISI-sum score n (%)	Not available	
No/subthreshold insomnia		26 (39)
Moderate insomnia		34 (51)
Severe insomnia		7 (10)
n		67 (target population)

^a^0-14 points: No/subthreshold insomnia, 15–21 points: moderate insomnia, 22–28: severe insomnia.

Source: Document analysis of patient data from the period January 2021–December 2021 of a top oncology center; own calculations.

### Sensitivity and specificity of the screening questions sleep disorder and tiredness

Table 3 indicates that for all parameters (sensitivity, specificity, positive predictive value (PPV), negative predictive (NPV)), the correlation was higher for the sleep disorder question than for the tiredness question. The differences were not statistically significant (Table 3).

**Table 3:** Parameters with representation of the p-values.

	Sleep disorder	Tiredness	p-values*
Sensitivity	0.804	0.725	0.179
Specificity	0.435	0.260	0.104
PPV	0.717	0.353	0.109
NPV	0.556	0.630	0.188

*p-values were calculated with a Monte-Carlo simulation using R.

Source: Document analysis of patient data from the period January 2021–December 2021 of a top oncology center; own calculations.

### Frequency analysis of response behavior to identify appropriate predictors of insomnia

First, the answers given by the patients at time t0 to the screening questions about sleep disorder and tiredness were cross-tabulated. As can be seen in Table 4, 44% of the participants show a coherent direction in their response behavior. They indicated that they suffer from relevant sleep disturbances as well as experience moderate to severe tiredness. In contrast, an inverse response behavior can be observed in 56% of the respondents: 30% reported moderate to severe tiredness despite no or little sleep disorders, whereas 26% of the participants seemed to be dominated by sleep disorders: these patients experience no to slight tiredness with the simultaneous presence of a problem falling or staying asleep or sleeping too much (Table 4).

**Table 4:** Distribution of sleep disorder and tiredness at time t0.

	Tiredness		N (%)
Sleep disorder	None/mild	Moderate/severe	
Not at all/several days	0	20 (30%)**	20 (30%)
More than half the days/nearly every day	17 (26%)**	29 (44%)*	46 (70%)
n	17	49	66

*Consistent response behavior.

**Inverse response behavior.

Source: Document analysis of patient data from the period January 2021–December 2021 of a top oncology center; own calculations.

Table 5 provides a more differentiated view of the symptom load. By cross-tabulating, the answers to the question about tiredness were compared to the degree of ISI values. Of all those patients with a follow-up appointment and ISI assessment at t1, 56% (35 of 63 patients) have a consistent response. For example, 10% (6 out of 63) of the participants had both no to mild tiredness and subthreshold insomnia, or 38% (24 out of 63) had moderate to severe tiredness as well as moderate insomnia. Conversely, this also means that a higher symptom load can be detected in 44% of the study participants, depending on the question. Based on the routine questioning during the follow-up (t1), 6 of 17 patients with no or slight tiredness (tiredness question) had subthreshold sleep disorder, 9 moderate and 2 severe insomnia (ISI sum index). Here it is clear that symptomatic insomnia could be detected in 11 of 17 patients in more differentiated questioning by means of ISI screening. At the same time, Table 5 also shows that 17 patients had only a subthreshold degree of insomnia despite a moderate to strong feeling of tiredness (Table 5).

**Table 5:** Distribution of tiredness and sleep disorder with the ISI sum score at time t1.

	Subtreshold insomnia	Moderate insomnia	Severe insomnia	N (%)
None/mild tiredness	6 (10%)*	9 (14%)**	2 (3%)**	17 (27%)
Moderate/severe tiredness	17 (27%)**	24 (38%)*	5 (8%)*	46 (73%)
n	23	33	7	63
Sleep disorder not at all/several days	10 (16%)*	8 (12%)**	0	18 (28%)
Sleep disorder more than half the days/nearly every day	13 (20%)**	26 (41%)*	7 (11%)*	46 (72%)
n	23	34	7	64

*Consistent response behavior.

**Inverse response behavior.

*Source*: Document analysis of patient data from the period January 2021–December 2021 of a top oncology center; own calculations.

To analyze a possible correlation between answering the question about sleep disorder and the degree of expression of the ISI surveyed afterward, again a cross-table was chosen for representation. 72% (46 of 64 patients) of the patients who responded to the sleep disorder question with the values more than half the days or nearly every day suffered from insomnia of varying severity: 20% subthreshold, 41% moderate, and 11% severe insomnia. Thus, in 52% of the participants, the existing symptom load was confirmed by the ISI sum score achieved in the following.

In total, 68% of the respondents agreed between the indication of trouble in falling or staying asleep or sleeping too much and the result of the ISI sum score, while 32% had deviations. If one compares these percentages with the above-mentioned values by answering the tiredness question, a more consistent response behavior to the sleep disorder question becomes apparent regarding the following ISI survey (Table 5).

### Mean comparison of the ISI sum score for the screening questions sleep disorder and tiredness

To approach the question of which instrument might be suitable for the detection of insomnia, the next step was to take a closer look at mean differences. In order to compare the groups with regard to the ISI sum score, the mean values were first calculated. Table 6 shows that patients who did not report any tiredness when answering the questionnaire showed a moderate level of insomnia when the ISI sum score was noted.

**Table 6:** Mean values of ISI sum score per inclusion criterion at time t1.

	Tiredness	
Sleep disorder	None/mild (n = 17)	Moderate/severe (n = 49)
Not at all or on some days (n = 20)		ISI: no/subthreshold insomnia (sum score 12)
On more than half the days or nearly every day (n = 46)	ISI: moderate insomnia (sum score 17)	ISI: moderate insomnia (sum score 17)

*Source*: Document analysis of patient data from the period January 2021–December 2021 of a top oncology center; own calculations.

To test whether the means of the ISI differed among the 3 groups:

- Inclusion based on tiredness
- Inclusion based on sleep disorder
- Inclusion based on both criteria

an ANOVA was performed.

The group, which was included solely based on the criterion of tiredness (n = 20), shows a significantly lower ISI total value (ISI sum score = 12) than the other two groups (inclusion based on sleep disorder (ISI sum score = 17) and inclusion based on both criteria (ISI sum score = 17).

Mean difference (D) between tiredness and sleep disorder (D = 4,541; 95% CI 1.69–7.39; p = 0.002) was significant as well as mean differences between sleep disorder and both criteria (D = 4,393; 95% CI 6.90–1.88; p = 0.001). Mean differences between tiredness and both criteria were not significant.

### Correlation analysis

**Figure 2:**
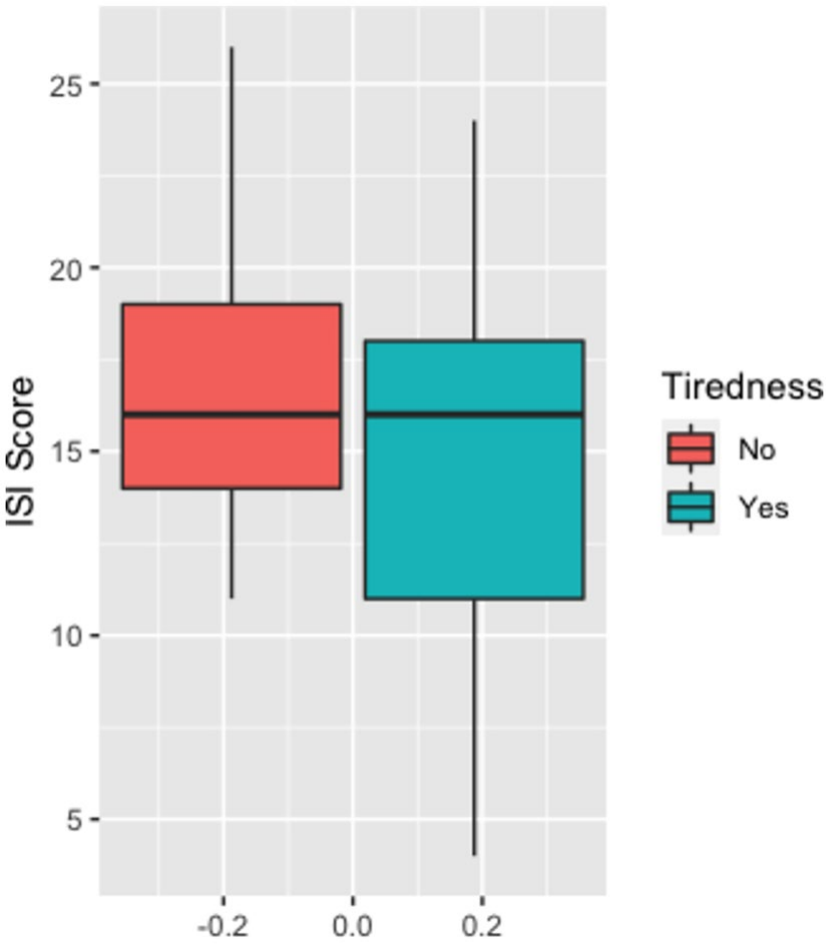
ISI sum score by tiredness boxplot. *Source*: Document analysis of patient data from the period January 2021–December 2021 of a top oncology center; own calculations.

To test whether there is a correlation between the information in the screening questions and the level of ISI, a linear correlation analysis was performed. The analysis indicated that sleep disorder was significantly related to the sum score of the ISI, with an effect size of r = 0.433 (p < 0.001). In contrast, no significant relationship was observed between reporting tiredness and the level of ISI (r = − 0.181, p = 0.146). The median for reporting no or mild tiredness is 16, as it is for moderate and severe tiredness (Fig. 2).

### Gender differences

**Figure 3:**
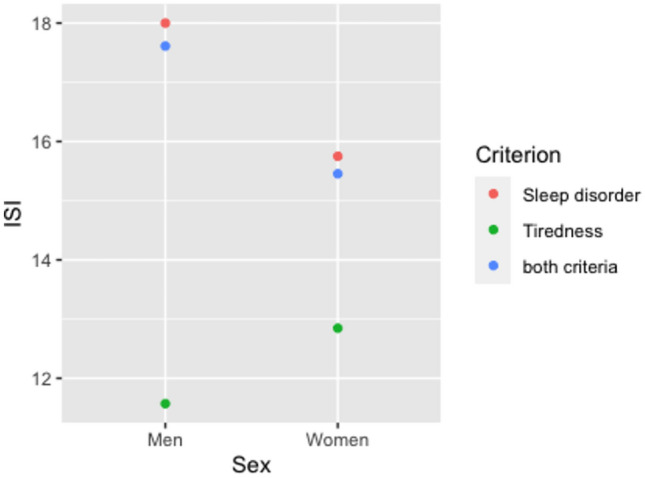
Gender-separated representation of the mean values of the ISI sum score per inclusion criterion. *Source*: Document analysis of patient data from the period January 2021–December 2021 of a top oncology center; own calculations.

Our analyses show biological sex-specific differences. Male patients stated less often tiredness than women and on the contrary a higher degree of sleep disorder or both than women. As can be seen in Fig. 3 the mean ISI sum score for men included because of the sleep disorder was 18, whereas among those included because of tiredness it was 11. Thus, the overall mean scores were further apart for men.

However, a significance analysis using two-factor ANOVA including gender and inclusion criteria showed no significant differences (p = 0.36). Additionally, t-tests were performed on the subgroups based on inclusion criteria. Neither for sleep disorder (t(15) = 1.200; p = 0.249) nor for tiredness (t(18) = 0.570; p = 0.576) nor both criteria (t(27) = 1.332; p = 0.194) significant gender differences could be found.

## Discussion

The high prevalence of insomnia among cancer patients and the associated impact on quality of life in everyday life underscores the importance of a comprehensive symptom assessment^42^. Given that sleep disorders and insomnia are often comorbid with somatic and psychiatric diseases in patients with cancer, the identification of appropriate predictors of insomnia in cancer patients is of particular importance^21^.

To our best knowledge, this is the first study, which extracts a screening question for insomnia out of a validated tool already used for cancer patients. The MIDOS^2^ and the PHQ-8 formed the basis for the assessment of patients with advanced cancer disease and sleep disorders. It became clear that patients who had a certain symptom burden when asked about sleep disorders (sleep disorders on more than half of the days or almost every day) showed higher sum scores in the subsequent questionnaire by the Insomnia Severity Index. Consequently, a sleep disorder question seems to be a positive predictor for the presence of insomnia and to be suitable as a screening question for cancer patients. In contrast, the assessment of tiredness is less suitable to detect insomnia. This is supported by the result of the correlation analysis, within which no significant relationship could be established between reported tiredness and the ISI sum score achieved. For example, patients with low tiredness had moderate insomnia in the subsequent ISI survey. Comparable results were obtained by a Canadian research group led by Savard^43^. By analyzing Canadian screening tools (ESAS-r and CPP) to identify patients with sleep disorders at an early cancer stage, they concluded that the addition of a specific sleep item was urgently needed to detect insomnia. Since in our study, mainly patients with advanced cancer stage were included, we agree with Savard’s claim also for this group.

We therefore suggest that patients who show a positive sleep disorder question should be assessed by ISI survey as soon as possible. As early as 2013, Howell et al. proposed the implementation of a two-stage screening procedure at the time of initial diagnosis and its continuation beyond the end of treatment^44^. However, the National Comprehensive Cancer Network (NCCN) Guidelines published in 2023 indicate that a significant proportion of cancer survivors are still receiving suboptimal care due to a lack of screening for sleep disorders^45^. A more detailed questioning of the sleep behavior would provide the treating physician with more precise information in advance in order to assess the patient’s level of suffering and give the possibility to deal with a sleep disorder and its accompanying symptoms more individually in the course of treatment. As there is currently no common in-depth screening for sleep disorders at German Oncology Centers, we aim to use our findings to highlight this situation and to encourage the implementation of insomnia screening for cancer patients. Our results appear to align with the algorithm proposed by the ESMO Clinical Practice Guideline, which suggests that if the box “sleep” is ticked in the Problem Check List, an assessment by the ISI should be carried out. If the score exceeds 15 points, a comprehensive assessment should be carried out in the form of a clinical interview. Lower scores mean re-screening at every follow-up visit^20^. The implementation of a close-meshed screening process could potentially reduce the risk of underdiagnosis of insomnia in everyday clinical practice. At the same time, it should be noted that a resource-conserving approach is particularly important for this vulnerable patient group. This also applies to the processing of the necessary screening instruments by the patient. Consequently, it was all the more important to use validated questions already implemented into the screening process to identify potential sleep disorders. As far as we know, little research has been done to date on the extent to which overtaxing and resulting non-acceptance could be a threat here. Studies in this regard would be beneficial.

Furthermore, the results show that especially in cancer patients tiredness can occur not only as a consequence of insomnia. The frequency analysis of the response behavior to the sleep disorder question and the tiredness question identified 20 patients (30%) with moderate to severe tiredness without relevant insomnia or sleep problems. This shows that tiredness in cancer patients can be caused by numerous factors: cancer-related anemia, psychological depression, cachexia, a loss of appetite, stressful medication (chemotherapeutic agents), obstructive sleep apnea syndrome (OSA), and listlessness are just a handful of these factors^21,46,47^. This constellation of symptoms may even make patients more prone to an increased need for sleep with daytime drowsiness, and support the aspect already mentioned above that the question about tiredness is not specific enough^48^. At this point, the role of OSA as an important cause of daytime sleepiness should be considered. With its high prevalence in the general population, OSA also affects cancer patients^21,49^. In particular, head and neck cancer and lung cancer patients often suffer from OSA^50,51^. Due to the potential association between cancer and OSA combined with underdiagnosis and negative effects on the quality of life of those affected, we agree with the requests for further investigations^52,53^.

A finely differentiated approach to the detection of insomnia therefore remains necessary in order to be able to assess and treat patients according to their needs, in particular because of the problem mentioned at the beginning that insomnia often remains undetected at first, sometimes due to the patients’ concern that a deviation from the treatment plan could be to the detriment of the cancer condition^1,12^. It would be beneficial for physicians to implement the ESMO Clinical Practice Guidelines, which recommends screening patients for insomnia^20^. Given the limitations of time and resources in everyday clinical practice, the use of validated questionnaires with a tablet at this point may be an effective tool in the oncological setting.

Initial approaches to gender differences suggest an initial underestimation of symptom burden in men by interviewing them using only single items about tiredness and sleep disorder. This statement can be reconciled with studies of gender-related patterns in symptom reporting. For example, there is repeated reference to differences in symptom perception between men and women, with women consistently tending to report subjective symptoms more frequently, which then leads to more frequent use of health services^54,55^. A qualitative study from 2021 that looked at gender differences in depression and burnout syndrome also concluded that men are often less open and more withdrawn and seek medical advice later. Early contact, especially with male patients, is therefore particularly important^56^. Thus, in order to elicit further clarification of exposure and a possible need for discussion specifically in men, the survey of the ISI could be more meaningful for men and therefore allow a more detailed recording of the initially indicated symptomatology.

### Limitations and potential for future research

At this point, it should be mentioned that this result can only be assessed to a limited extent due to the lack of inclusion of patients who exclusively reported no and mild tiredness without relevant falling asleep or staying asleep issues. Group-specific and gender-specific differences should be interpreted with caution, since a very specific group with a small number of cases was analyzed. This leads to an over- or underestimation of associations in each case. Furthermore, the single-center design of this study may have resulted in a potential selection bias, which could limit the generalizability of the findings. It may be beneficial for future research to consider expanding the sample size and focusing on patients with only a positive sleep disorder question.

A multi-center study using all screening instruments and systematically testing group differences, especially as our results generally between insomnia assessment tools show incongruence and gender differences, would be an evidence-based step towards setting jointly agreed standards for the systematic assessment of insomnia in cancer patients.

## Conclusion

Due to the frequency of insomnia in cancer patients and the suffering often associated with it, identification of those affected should take place as early as possible in order to provide comprehensive support services. For an initial assessment, the sleep disorder question about falling or staying asleep or sleeping too much seems to be suitable as a positive predictor for the presence of insomnia. To raise awareness of the relevance and progression of insomnia in cancer patients, an easy-to-implement measure would be to help patients through an additional survey of ISI to capture the complex symptomatology. One possible integration of our results into everyday clinical practice could be that, in instances where the sleep disorder question is answered “on more than half the days” or “nearly every day”, the patient would be directly offered the chance to complete the ISI on a tablet. Especially as patients and digitalized hospitals already have equipment (tablets) and experiences in this area.

## Data Availability

Data is available from the corresponding author on reasonable request.
